# Method Evaluation of the QuidelOrtho Diagnostics Vitros NT-proBNP II Assay

**DOI:** 10.3390/jcm13247751

**Published:** 2024-12-19

**Authors:** Yi Xiao, Chao Sun, Justin Tsan, Edward Ki Yun Leung

**Affiliations:** 1 Department of Pathology and Laboratory Medicine, Children’s Hospital Los Angeles, 4650 Sunset Blvd, M/S #32, Los Angeles, CA 90027, USA; yxiao@chla.usc.edu (Y.X.); chasun@chla.usc.edu (C.S.); jtsan@chla.usc.edu (J.T.); 2 Department of Pathology, University of Southern California Keck School of Medicine, Los Angeles, CA 90027, USA

**Keywords:** method evaluation, NT-proBNP, QuidelOrtho

## Abstract

**Background/Objectives:** N-terminal-proBNP (NT-proBNP) is a biomarker released into the blood in response to heart failure, reflecting the extent of cardiac stress and damage. QuidelOrtho Diagnostics released its latest reformulation of its NT-proBNP assay, the Vitros NT-proBNP II assay. This study aims to evaluate the analytical performance of the Vitros NT-proBNP II assay. **Methods**: Repeatability, reproducibility, carryover, analytical measurement range, and clinical reportable range (AMR and CRR) were assessed using commercially available materials and dilution of patient specimens. Accuracy was evaluated by comparing results from the Vitros NT-proBNP II and the Vitros NT-proBNP assays. Paired heparin and EDTA plasma specimen results were compared, and instrument-to-instrument comparison was performed using two different Vitros 5600 Integrated Systems. NT-proBNP stability was evaluated at room temperature, 2–8 °C, and −18 °C for up to five days. **Results**: Repeatability and reproducibility were ≤10% CV, and no carryover was observed. The AMR was 20–30,000 pg/mL and dilution up to 80 times was verified. Passing–Bablok analysis showed a significant proportional bias with a slope of 1.37. Instrument-to-instrument and heparin-to-EDTA plasma comparisons showed no significant biases. NT-proBNP is stable up to five days at room temperature, 4 °C, and −20 °C. **Conclusions**: Our evaluation demonstrated acceptable analytical performances of the Vitros NT-proBNP II assay except for the positive proportional bias compared with the Vitros NT-proBNP assay.

## 1. Introduction

Congestive heart failure (CHF) is a complex condition that occurs when the heart’s ability to pump blood is impaired, resulting in inadequate blood flow to meet the body’s needs. CHF affects approximately 64 million people worldwide [1], and the core issue is ventricular dysfunction, often indicated by a reduced cardiac ejection fraction (EF) [2]. Heart failure is typically diagnosed when the EF drops to 40% [2].

Before the discovery of brain natriuretic peptide (BNP) in 1988 [3], the diagnosis of heart failure relied primarily on clinical symptoms and tools like echocardiography and X-rays, without the use of biochemical markers [4]. BNP is later found to be secreted in large amounts when there is an increase in intracardiac blood volume or left ventricular pressure overload [5]. Ventricular muscle and brain cells can produce a precursor to BNP known as pre-proBNP, which consists of 134 amino acids [6]. Within the cells, the signal peptide is removed, resulting in the formation of a 108-amino-acid peptide called proBNP, which is then released into the bloodstream [6]. Once in the blood, proBNP is further broken down by enzymes into two separate peptides: a 32-amino-acid B-type natriuretic peptide (BNP) and a 76-amino-acid N-terminal proBNP (NT-proBNP) [6]. The biological functions of BNP include increasing natriuresis, enhancing diuresis, and reducing blood volume [5]. Although the biological effects of NT-proBNP are largely unknown, it still reflects BNP secretion levels [7]. Serum or plasma concentrations of BNP and NT-proBNP are the best single assessments for predicting and diagnosing heart failure, with high negative predictive values [2,8]. They are useful for confirming or ruling out heart failure in patients with atypical signs and symptoms in both acute and non-acute conditions [2]. Additionally, they can help differentiate between chronic heart failure with dyspnea and lung disease [9]. However, other conditions causing water and sodium retention, leading to increased blood volume, can also elevate BNP levels. Such conditions include primary aldosteronism [10], liver cirrhosis [11], and renal failure [7].

NT-proBNP testing has several advantages over BNP testing. The half-life of BNP is around 20 min, while that of NT-proBNP is around 120 min [12]. This longer half-life results in higher concentrations of NT-proBNP in the circulation of heart failure patients, typically 1 to 10 times greater than BNP concentrations [13,14], thus NT-proBNP is more sensitive for the diagnosis and monitoring of heart failure.

Furthermore, compared with BNP, NT-proBNP is less susceptible to interferences from heart failure therapies like Nesiritide and angiotensin receptor-neprilysin inhibitors (ARNI) [15,16,17]. Nesiritide, a synthetic recombinant form of BNP, mimics the effects of endogenous BNP. Its administration artificially increases BNP levels but does not affect NT-proBNP, making NT-proBNP a better marker in patients receiving Nesiritide [17]. Similarly, ARNI therapy inhibits neprilysin, an enzyme responsible for degrading BNP, leading to elevated BNP levels after treatment [18]. In contrast, NT-proBNP is cleared primarily through the kidneys and is unaffected by neprilysin [18]. This difference in clearance also explains why NT-proBNP has a longer half-life in serum compared to BNP.

NT-proBNP can be measured by enzyme-linked immunoassay, chemiluminescence, and electrochemiluminescence immunoassay. Commercialized kits such as the Roche Elecsys NT-proBNP and QuidelOrtho Vitros NT-proBNP have been developed. Measuring NT-proBNP presents several challenges. First, some immunoassays only recognize the mid-fragment of the NT-proBNP molecule, but NT-proBNP is often glycosylated at nine distinct sites [19]. This may lead to underestimation of NT-proBNP levels if the middle region is glycosylated [20]. Additionally, NT-proBNP immunoassays are not standardized, and different peptides used for calibration made it difficult for comparisons [21]. Furthermore, several NT-proBNP assays, including earlier OrthoQuidel Vitros assays, may cross-react with proBNP [22]. According to the manufacturer, the newly-introduced QuidelOrtho Vitros NT-proBNP II test offers several advantages over the older Vitros NT-proBNP assay, including less interference from biotin, a longer calibration interval, and lower limit of quantitation (10 pg/mL for the Vitros NT-proBNP assay versus 0.56 pg/mL for the Vitros NT-proBNP II assay) [23,24]. This study aims to evaluate the analytical performance of the Vitros NT-proBNP II assay.

## 2. Materials and Methods

### 2.1. Chemicals and Materials

Reagents, calibrators, and high sample diluent B for the Vitros NT-proBNP and NT-proBNP II assays were purchased from QuidelOrtho (San Diego, CA, USA). Quality Control (QC) materials were purchased from Bio-rad laboratories (Hercules, CA, USA). Linearity and calibration verification materials were purchased from LGC Clinical Diagnostics (Milford, MA, USA). Leftover patient K_2_EDTA and lithium heparin plasma specimens, as well as serum specimens that otherwise would be discarded, were obtained from the core laboratory of the Department of Pathology and Laboratory Medicine at Children’s Hospital Los Angeles (CHLA).

### 2.2. Measurement of NT-proBNP

Both the Vitros NT-proBNP and NT-proBNP II chemiluminescent immunometric assays were performed on the VITROS 5600 Integrated Systems from QuidelOrtho using the VITROS^®^ MicroWell technology. The measurement process involves both incubating the patient specimen with a horseradish peroxidase (HRP)-labeled NT-proBNP detection antibody and a biotinylated NT-proBNP capture antibody. With the capture antibody and detection antibody bound to different epitopes of NT-proBNP, a sandwich antibody–antigen–antibody complex is formed, which is captured by Streptavidin coated microwells. Unbound materials are removed by washing, and a reagent containing a luminogenic substrate and an electron transfer agent is then added. The luminogenic substrate is oxidized by HRP to generate light, with intensity further amplified by the electron transfer agent. The generated light signals are measured, which are directly proportional to the concentration of NT-proBNP in the specimen. Details on the differences between the Vitros NT-proBNP and NT-proBNP II assays are not disclosed.

### 2.3. Validation Procedures

Precision studies were performed using quality control (QC) materials on two different Vitros 5600 Integrated Systems (i.e., Vitros A and Vitros B). Repeatability (intra-day precision) and carryover were assessed by performing pentaplicate measurements of two different levels of QC materials in an alternating pattern to obtain a total of 20 data points per QC level, all collected within one run on the same day. Reproducibility (inter-day precision) was assessed by measuring each level of QC materials daily over 20 different days. Analytical measurement range (AMR) was evaluated with duplicated analysis of the linearity and calibration verification materials. Clinical reportable range (CRR) was evaluated by assessing the recoveries after diluting patient specimens using high sample diluent B.

Accuracy was evaluated by comparing results from the Vitros NT-proBNP and NT-proBNP II assays using patient K_2_EDTA plasma specimens. An instrument-to-instrument comparison was also performed by analyzing the same plasma specimens on two different Vitros 5600 Integrated Systems. A specimen-type comparison was performed by analyzing paired lithium heparin and K_2_EDTA plasma specimens using the Vitros NT-proBNP II assay. Results from accuracy assessment, instrument comparison, and specimen-type comparison studies were analyzed using Passing–Bablok linear regression and Bland–Altman bias plot analysis. Specimen stability was assessed by measuring pooled patient specimens aliquoted and stored at room temperature, 4 °C, and −20 °C. Duplicate measurements were performed for each aliquot stored at each temperature over a period of 5 days. Recoveries from each storage condition were calculated.

Centrifugation of separated plasma specimens were performed using a StatSpin Express 4 high-speed Horizontal Centrifuge at 4000× *g* for 5 min.

Statistical analyses (i.e., Passing–Bablok and Bland–Altman analyses) were performed using Analyse-it (Analyse-it Software Ltd., Leeds, UK, Version 5.10.9). Results from the AMR study were plotted using GraphPad Prism (GraphPad Software, San Diego, CA, USA, Version 8.4.3).

## 3. Results

### 3.1. Precision, AMR, and CRR

**Figure 1:**
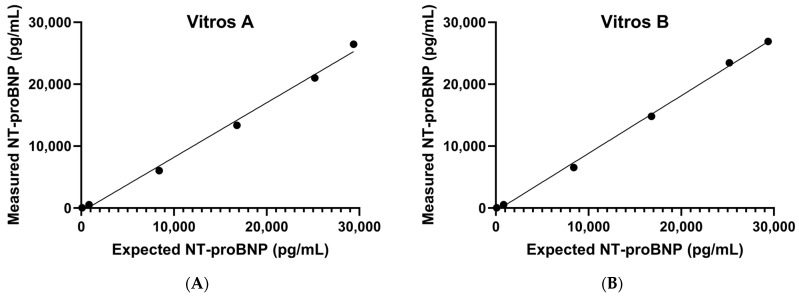
The claimed analytical measurement range of 20–30,000 pg/mL was verified on two Vitros 5600 Integrated Systems, Vitros A (**A**) and Vitros B (**B**).

The repeatability coefficient of variations (CVs) ranged from 2% to 5%, and reproducibility CVs ranged from 3% to 10% (Table 1). No carryover effect was observed. The claimed AMR of 20–30,000 pg/mL from QuidelOrtho was verified (Figure 1A,B). Dilutions up to 80 times were verified to extend the CRR to up to 600,000 pg/mL using the high sample diluent B to cover specimens with very elevated NT-proBNP concentrations, with recoveries ranging from 90% to 110%.

### 3.2. Accuracy

**Figure 2:**
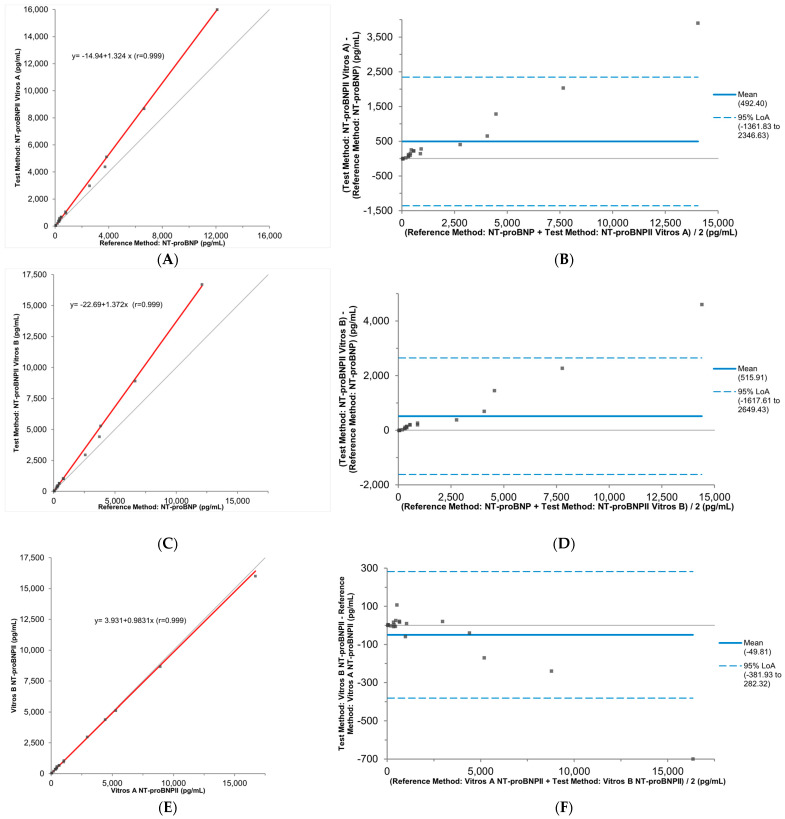
Results from method comparison studies analyzed with Passing–Bablok regression and Bland–Altman plots. Positive proportional biases were observed when comparing results from the Vitros NT-proBNP II assay using the Vitros A and B analyzers with results from the Vitros NT-proBNP assay (**A**–**D**). Results from both Vitros 5600 Integrated Systems were comparable (**E**,**F**). In the Passing–Bablok plots, regression lines are plotted in red. In the Bland–Altman plots, the mean average is represented by the blue solid line, and the 95% Limits of Agreement (LoA) is represented by the blue dashed lines. In both the Passing–Bablok plots and the Bland–Altman plots, the identify line indicating no differences between the test and reference methods is plotted in grey.

Accuracy was evaluated by measuring each K_2_EDTA plasma specimen using the Vitros NT-proBNP and NT-proBNP II assays within a few hours of each other. Both Passing–Bablok and Bland–Altman analyses revealed a significant and unexpected positive proportional bias when comparing results from the Vitros NT-proBNP II assay with those from the Vitros NT-proBNP assay (Vitros A: slope = 1.32, 95%CI: 1.21–1.52; y-intercept = −15 pg/mL, 95%CI: −32–33 pg/mL; mean bias = 492 pg/mL, 95%CI: 50–935 pg/mL; r = 0.99; *n* = 23. Figure 2A,B). A similar positive proportional bias was also observed when the Vitros NT-proBNP II Assay was performed on the other Vitros 5600 integrated System (Vitros B: slope = 1.37, 95%CI: 1.27–1.41; y-intercept = −23 pg/mL, 95%CI: −37–−3 pg/mL; mean bias = 516 pg/mL, 95%CI: 20–1011 pg/mL; r = 0.99; *n* = 23, Figure 2C,D). NT-proBNP II results from both Vitros 5600 integrated systems were comparable (slope = 0.98, 95%CI: 0.96–1.01; y-intercept = 4 pg/mL, 95%CI: 0–30 pg/mL; mean bias = −50 pg/mL, 95%CI: −129–30 pg/mL; r = 0.99; *n* = 23, Figure 2E,F).

The positive bias cannot be reduced after dilution with high sample diluent B (*n* = 4) (Table 2). Furthermore, the positive bias persists following double centrifugation at 4000× *g* for 5 min, following one freeze–thaw cycle, and following one freeze–thaw cycle followed by a single centrifugation at 4000× *g* for 5 min (Table 3). The positive bias is also found in both K_2_EDTA plasma and serum specimens (Table 3). According to QuidelOrtho, both serum and plasma specimens for the Vitros NT-proBNP II assay can be stored for up to 26 weeks when frozen and up to three freeze–thaw cycles [24]. However, data from one of the tested serum specimens suggests that NT-proBNP values are significantly reduced after one freeze–thaw cycle (Recovery = 79%, specimen ID # 7) (Table 3).

### 3.3. Stability and Specimen Type Comparison

**Figure 3:**
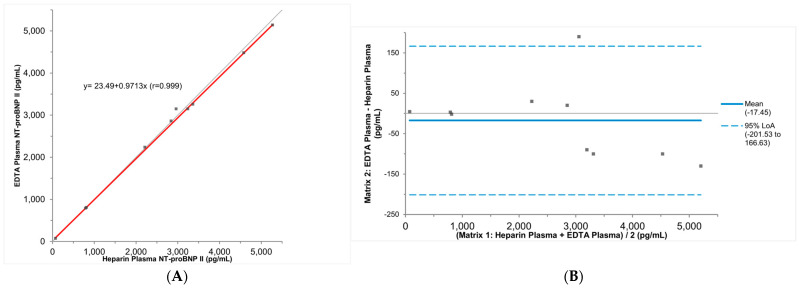
Results from the specimen-type comparison study analyzed with Passing–Bablok regression (**A**) and Bland–Altman plot (**B**). In the Passing–Bablok plots, regression lines are plotted in red. In the Bland–Altman plots, the mean average is represented by the blue solid line, and the 95% Limits of Agreement (LoA) is represented by the blue dashed lines. In both the Passing–Bablok plots and the Bland–Altman plots, the identify line indicating no differences between the test and reference methods is plotted in grey.

Pooled K_2_EDTA plasma specimens stored at room temperature, 4 °C, and −20 °C for up to five days remained stable when measured using the Vitros NT-proBNP II assay, with recoveries ranging from 90% to 110%. Passing–Bablok and Bland–Altman analyses of results from paired K_2_EDTA plasma and lithium heparin plasma showed comparable results (slope = 0.97, 95% CI: 0.94–1.01; y-intercept = 23 pg/mL, 95% CI: 0–189; mean bias = −50 pg/mL, 95% CI: −84–50 pg/mL; *n* = 10, Figure 3A,B).

## 4. Discussion

Reformulation of assays by manufacturers is not uncommon, either to accommodate changes in how ingredients and parts are sourced, or to address the need to improve assay performance. The motivation for us to perform this study is simply to verify the claimed analytical performance of the reformulated Vitros NT-proBNP assay. The analytical evaluation of the Vitros NT-proBNP II assay demonstrated acceptable precision and the vendor’s measurement range was verified. However, a significant and unexpected positive proportional bias was identified when compared with the Vitros NT-proBNP assay, which was not consistent with the official data published by QuidelOrtho [25].

To investigate the root cause, we considered and eliminated several potential factors. Initially, we used results from the Vitros NT-proBNP assay measured several days prior for the accuracy study. One might reasonably suspect that NT-proBNP had degraded into multiple fragments, each of which could separately react with the capture and detection antibodies, leading to the observed positive bias. To address this concern, each specimen included in the accuracy study was analyzed using both the Vitros NT-proBNP and NT-proBNP II assays within a few hours of each other. Despite this, the NT-proBNP II assay continued to show a significant positive bias.

We also investigated whether the positive bias could be reduced or eliminated through dilution, a property typically associated with the presence of an interfering compound. The persistence of the positive bias up to a 1:10 dilution of the original specimen suggests that an interfering compound is unlikely (Table 2).

We also investigated if the positive bias is caused by storage of the specimens at −20 °C and the formation of microclots. To explore this possibility, we aliquoted fresh patient K_2_EDTA plasma specimens (*n* = 2) and measured them without any treatment, after double centrifugation at 4000× *g* for 5 min, after one freeze–thaw cycle, and after one freeze–thaw cycle followed by a single centrifugation at 4000× *g* for 5 min. Despite these efforts, the positive biases from the Vitros NT-proBNP II assay persisted (Table 3).

According to QuidelOrtho, serum is considered an acceptable specimen type. Although we did not conduct a study to verify serum as an alternative specimen type, the positive bias from the Vitros NT-proBNP II assay was observed in serum specimens (*n* = 2), with the level of bias comparable to that seen in plasma specimens (Table 3). Only two serum specimens were included in the study, and recovery from one specimen after one freeze–thaw cycle was 79%, inconsistent with what is claimed in the package insert [24].

Accuracy was evaluated by measuring K_2_EDTA plasma specimens using the Vitros NT-proBNP and NT-proBNP II assays within a few hours of each other. Both Passing–Bablok and Bland–Altman analyses revealed a significant and unexpected positive proportional bias when comparing results from the Vitros NT-proBNP II assay with those from the Vitros NT-proBNP assay, with insignificant constant bias. Unfortunately, the positive proportional bias cannot be resolved by assay re-calibration. Gaussian distribution was identified from the percentage biases, and regression analysis between the percentage biases and NT-proBNP results showed no significant correlation (*p* = 0.53). The 95% limits of agreement ranges from −14% to 56%, suggesting a wide variation of the percentage difference between the Vitros NT-proBNP and NT-proBNP II test.

**Figure 4:**
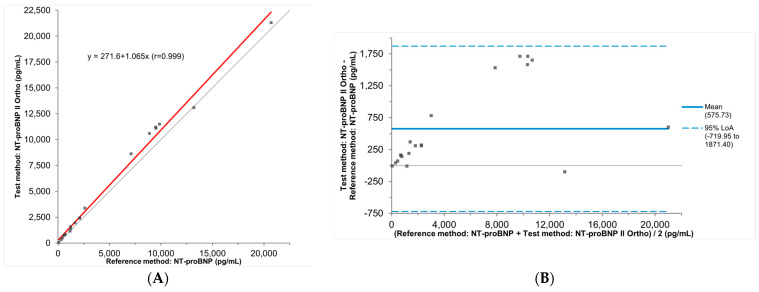
Results from QuidelOrtho’s method comparison study between both the Vitros NT-proBNP and NT-proBNP II assays. With unweighted Deming regression analysis performed by the vendor (**A**), no proportional bias was found. However, a closer analysis of the Bland–Altman plot (**B**) showed that the statistical analysis was skewed by the two very elevated results exceeding 13,000 pg/mL. In the Passing–Bablok plots, regression lines are plotted in red. In the Bland–Altman plots, the mean average is represented by the blue solid line, and the 95% Limits of Agreement (LoA) is represented by the blue dashed lines. In both the Passing–Bablok plots and the Bland–Altman plots, the identify line indicating no differences between the test and reference methods is plotted in grey.

We then notified and consulted QuidelOrtho. Both Vitros 5600 Integrated Systems were inspected by a QuidelOrtho field service engineer and were verified to be working as intended. Additionally, a QuidelOrtho field application scientist confirmed that both the Vitros NT-proBNP and NT-proBNP II assays were configured correctly. As part of the investigation, the field application scientist performed an independent study using leftover serum and K_2_EDTA plasma specimens analyzed by both assays and statistical analysis of this data using unweighted Deming regression (slope = 1.07, 95%CI: 1.01–1.11; y-intercept = −272 pg/mL, 95%CI: 61–530 pg/mL; mean bias = 578 pg/mL, 95%CI: −720–1871 pg/mL; r = 0.99; *n* = 20, Figure 4A), which did not show a significant positive proportional bias. Based on this data, QuidelOrtho concluded the instruments and assays were performing within the expected parameters. However, a closer analysis of the Bland–Altman plot (Figure 4B) showed the statistical analysis was skewed by the two highly elevated results exceeding 13,000 pg/mL, where less significant bias was observed. Analysis of the individual paired data results showed significant positive biases for lower values, including those close to the reference range, measured on the NT-proBNP II assay. The inclusion of those two highly elevated NT-proBNP values masked the significant positive biases observed in lower concentrations, where results are more clinically relevant. This underscores the importance of carefully reviewing and scrutinizing the validation data and results, rather than relying only on statistical analysis alone. Not all biases are independent of analyte concentrations, and concentration-dependent biases may be more easily identified from the Bland–Altman plot.

Given the significant positive proportional bias with minimal constant bias, adjustment of the reference range or the use of a correction factor may be considered and investigated before implementing the Vitros NT-proBNP II assay. The most commonly used cut-off value for NT-proBNP is 125 pg/mL, with values below this threshold used to rule out heart failure [26,27]. This cut-off was proposed as part of the universal definition of heart failure [26]. Any positive proportional bias seen in the new Vitros NT-proBNP assays could inadvertently prompt patients to undergo unnecessary workup for heart failure. When NT-proBNP is used for severity tiering, this positive bias may result in overestimation of the disease’s severity. While a correction factor or adjustment of the reference range could be attempted to address the bias, it would not resolve the underlying cause. Moreover, adjustment of the reference range based on a single data set may not generalize well across different patient populations or clinical settings.

Adjusting reference ranges for proportional bias can also make it difficult to compare current data with historical data. This can be problematic in long-term patient monitoring. Providing consistent and analytically acceptable results is particularly important for clinicians who use the assay to trend patient results over time. However, without reference range adjustments or correction factors, results from the two different assays could also lead to an abrupt shift in reported values.

## 5. Conclusions

Our evaluation demonstrated acceptable analytical performances of the Vitros NT-proBNP II assay except for the positive proportional bias compared with the Vitros NT-proBNP assay. Based on our data, adjustment of the reference range or the use of a correction factor may be considered and investigated before implementing the Vitros NT-proBNP II assay.

## Figures and Tables

**Table 1:** Results from precision studies for the Vitros NT-proBNP II assay.

		Repeatability (*n* = 20)		Reproducibility (*n* = 20)	
		Mean (pg/mL)	CV (%)	Mean (pg/mL)	CV (%)
Vitros A	QC1	48	4	45	5
	QC2	2895	2	2829	3
Vitros B	QC1	51	5	47	10
	QC2	3015	2	2825	6

**Table 2:** The positive bias from the Vitros NT-proBNP II assay cannot be reduced by dilutions using high sample diluent B (*n* = 4).

Specimen ID #	Dilution	Results from the Vitros NT-proBNP Assay (pg/mL). Results from Duplicated Measurements Are Documented When Available		Results from the Vitros NT-proBNP II Assay (pg/mL). Results from Duplicated Measurements Are Documented When Available		% Recovery of the Result (Vitros NT-proBNP II Assay vs. Vitros NT-proBNP Assay). Results Are Averaged When Duplicated Measurements Were Performed
1	Neat	16,400		18,800		115%
	1:4	4130	4090	4740	4760	116%
	1:10	1470	1480	1730	1700	116%
2	Neat	9910		11,000		111%
	1:4	2460	2440	2710	2730	111%
	1:10	919	926	1050	1030	113%
3	Neat	2590		3350		129%
	1:4	667	661	798	824	122%
	1:10	274	270	324	322	119%
4	1:4	2470	2470	2750	2790	112%
	1:10	945	937	1040	964	106%

**Table 3:** The positive bias from the Vitros NT-proBNP II assay cannot be reduced by (1) double centrifugation at 4000× *g* for 5 min, or (2), one freeze–thaw cycle, or (3) one freeze–thaw cycle followed by a single centrifugation at 4000× *g* for 5 min.

Specimen ID #	Sample Type	Sample Preparation	Results from the Vitros NT-proBNP Assay (pg/mL)	Results from the Vitros NT-proBNP II Assay (pg/mL)	% Recovery of the Result (Vitros NT-proBNP II Assay vs. Vitros NT-proBNP Assay)
5	K_2_EDTA plasma	N/A	4350	5650	130%
		Double centrifugation	4290	5640	131%
		One freeze–thaw cycle	4300	5570	130%
		One freeze–thaw cycle with a single centrifugation at 4000× *g* for 5 min	4410	5670	129%
6	K_2_EDTA plasma	N/A	5320	6210	117%
		Double centrifugation	5320	6510	122%
		One freeze–thaw cycle	5200	6410	123%
		One freeze–thaw cycle with a single centrifugation at 4000× *g* for 5 min	5240	6430	123%
7	Serum	N/A	7100	8630	122%
		One freeze–thaw cycle	5760	6840	119%
8	Serum	N/A	1220	1590	130%
		One freeze–thaw cycle	1120	1480	132%

## Data Availability

The data presented in this study are available on request from the corresponding author because the data was collected for internal use at Children’s Hospital Los Angeles for verification studies.
